# Autocatalytic effect boosts the production of medium-chain hydrocarbons by fatty acid photodecarboxylase

**DOI:** 10.1126/sciadv.adg3881

**Published:** 2023-03-31

**Authors:** Poutoum P. Samire, Bo Zhuang, Bertrand Légeret, Ángel Baca-Porcel, Gilles Peltier, Damien Sorigué, Alexey Aleksandrov, Frédéric Beisson, Pavel Müller

**Affiliations:** ^1^ Aix-Marseille University, CEA, CNRS, Institute of Biosciences and Biotechnologies, BIAM Cadarache, 13108 Saint-Paul-lez-Durance, France.; ^2^ Université Paris-Saclay, CEA, CNRS, Institute for Integrative Biology of the Cell (I2BC), 91198 Gif sur Yvette, France.; ^3^ LOB, CNRS, INSERM, Ecole Polytechnique, Institut Polytechnique de Paris, 91128 Palaiseau Cedex, France.

## Abstract

Ongoing climate change is driving the search for renewable and carbon-neutral alternatives to fossil fuels. Photocatalytic conversion of fatty acids to hydrocarbons by fatty acid photodecarboxylase (FAP) represents a promising route to green fuels. However, the alleged low activity of FAP on C2 to C12 fatty acids seemed to preclude the use for synthesis of gasoline-range hydrocarbons. Here, we reveal that *Chlorella variabilis* FAP (*Cv*FAP) can convert *n*-octanoic acid in vitro four times faster than *n*-hexadecanoic acid, its best substrate reported to date. In vivo, this translates into a *Cv*FAP-based production rate over 10-fold higher for *n*-heptane than for *n*-pentadecane. Time-resolved spectroscopy and molecular modeling demonstrate that *Cv*FAP’s high catalytic activity on *n*-octanoic acid is, in part, due to an autocatalytic effect of its *n*-heptane product, which fills the rest of the binding pocket. These results represent an important step toward a bio-based and light-driven production of gasoline-like hydrocarbons.

## Teaser

Gasoline-like hydrocarbons can be efficiently produced by a natural algal photoenzyme using nothing but fatty acids and sunlight.

## INTRODUCTION

Fatty acid photodecarboxylase (FAP; EC 4.1.1.106), a member of the glucose-methanol-choline oxidoreductase family, is an algae-specific enzyme harboring a flavin adenine dinucleotide (FAD) cofactor (*1*). FAP is one of the few known natural photoenzymes besides photosynthetic reactions centers (which are protein complexes), DNA-repairing enzymes photolyases (*2*), and light-dependent protochlorophyllide oxidoreductases (*3*). Despite the short time since the discovery of FAP in the green microalga *Chlorella variabilis* by Sorigué *et al.* (*1*), numerous groups have already explored the potential applications of this enzyme in biocatalytic processes. *C. variabilis* FAP (*Cv*FAP) represents a new, attractive, light-driven, and redox-neutral means for the production of *n*-alkanes and *n*-alkenes as a basis for fuels, chemistry, and cosmetics (*4*–*7*). *Cv*FAP also appears to be a very promising tool for the photocatalytic synthesis of specialty chemicals such as deuterated hydrocarbons (*8*), enantiomerically pure α-amino acids, α-hydroxy acids (*9*), secondary fatty alcohols (*10*), and aliphatic amines and esters (*11*).

The natural substrates for the photoproduction of hydrocarbons in the green algae *C. variabilis* and *Chlamydomonas reinhardtii* were previously shown to be C16 to C18 linear fatty acids (FAs) (*12*). Following the discovery of FAP, an initial in vitro characterization of purified *Cv*FAP and its *Chlamydomonas* homolog indicated that these enzymes exhibited higher affinity for longer (C16 to C18) FAs compared to C12 to C14 FAs (*1*). It was also found that the crystal structure of *Cv*FAP expressed in the heterologous host *Escherichia coli*, which contains C10 to C18 FAs, had two “native” (likely unsaturated) C18 FA substrates—one at the active site and another one stabilized at the surface of the protein close to the entrance to the tunnel leading to the active site (*13*). In addition, expression in *E. coli* of four other FAPs chosen in different algal groups among almost 200 putative FAPs identified in algae genomes or metagenomic data showed that they were preferentially performing photodecarboxylation of the endogenous C16 to C18 FAs rather than C10 to C14 FAs (*14*). Use of *E. coli* cell-free extracts expressing the enzyme confirmed the preference of *Cv*FAP for C16 to C18 FAs over C12 to C14 FAs (*4*) and showed that it had low activities on C2 to C6 FAs (*15*). Together, all these data lead to the view that *Cv*FAP was adapted to act on C16 to C18 substrates and was not likely to be an efficient biological catalyst to produce gasoline-range (C5 to C11) hydrocarbons.

Here, we reveal that, under the right conditions, *Cv*FAP can be highly active on C8 to C10 medium-chain FAs in vitro. We also show that the high activity of *Cv*FAP on C8 to C10 FAs can be attributed in part to an unexpected autocatalytic effect, for which we provide spectroscopic evidence and molecular modeling support. Last, a bioconversion experiment using bacterial cultures expressing *Cv*FAP provides evidence that this biocatalyst can be used to produce medium-chain hydrocarbons in a much more efficient way than long-chain hydrocarbons.

## RESULTS

### Spectroscopic estimation of C7 to C18 FA decarboxylation quantum yield and the observation of autocatalysis

To understand the previously reported preference of *Cv*FAP for C16 to C18 FAs over shorter ones, we decided to screen the yield of the initial photochemical steps associated with decarboxylation of saturated C7 to C18 FA substrates using time-resolved fluorescence (TRF) and transient absorption spectroscopy (TAS). As shown previously (*1*, *13*), the photoexcited singlet FAD cofactor (^1^FAD*) of *Cv*FAP is fluorescent, and in the absence of a substrate, its fluorescence decays with a time constant of ~5 ns, yielding an FAD triplet (^3^FAD*), which reverts to the ground-state FAD with a time constant of ~80 μs (*1*). However, in the presence of a deprotonated FA substrate (R-COO^−^) in the active site, the ^1^FAD* fluorescence is quenched by forward electron transfer (ET) from the substrate to the ^1^FAD*, yielding an FAD^∙−^ anion radical, an alkyl radical R^∙^, and CO_2_ (*13*). This is reflected by the appearance of a fast (~300 ps) phase in the TRF signal. The ratio of the amplitudes of the two phases then directly reflects the ratio of the two processes, the unproductive intersystem crossing of ^1^FAD* to ^3^FAD* in ~5 and the productive ET from the FA substrate to ^1^FAD* in ~300 ps, which is followed by a quasi-instantaneous substrate decarboxylation (*13*). Given that decarboxylation appears to be essentially irreversible (*13*), we suggest that the share of the ~300-ps phase in the TRF signal roughly corresponds to the photodecarboxylation quantum yield.

The subsequent photoreaction step (back ET from FAD^∙−^ to the alkyl radical R^∙^ in ~100 ns) leads to the reoxidized flavin with a transiently red-shifted absorption spectrum, FAD_RS_ [see figure S6C in (*1*) for FAD_RS_ − FAD difference spectrum in solution or figure S12 (A and B) in (*13*) for cryo-trapped FAD_RS_]. The formation (and decay) of FAD_RS_ is best followed by monitoring transient absorption changes around 520 nm (maximum in the FAD_RS_ − FAD difference spectrum) (*1*, *13*). At room temperature, the red shift disappears within ~3 ms, likely upon binding of a new substrate and/or restoration of the initial charge distribution and hydrogen bonding network around FAD (*1*, *13*).

**Fig. 1.**
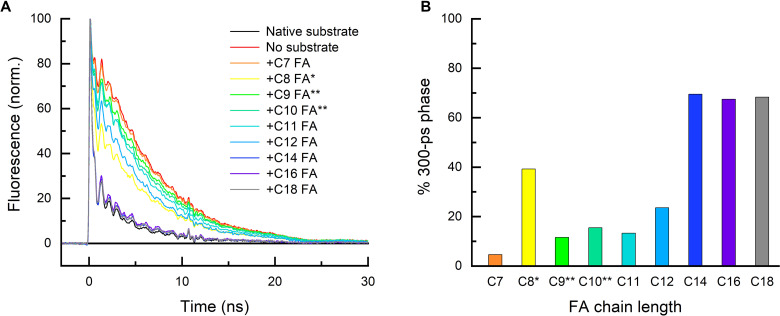
Photodecarboxylation of saturated linear C7 to C18 fatty acids (FAs) by *Cv*FAP. (**A**) Normalized time-resolved fluorescence (TRF) signals at 560 nm recorded for ~30 μM *Cv*FAP with the native substrate before its consumption, after its consumption (no substrate), and with added ~300 μM C7 to C18 FA substrates (see legend for color code). Most of the latter signals were recorded after five strong (~10 mJ/cm^2^) 470-nm flashes, i.e., after almost complete consumption of the strongly bound native substrate (see fig. S1). The signals for C8, C9, and C10 FAs are those for which the amplitude of the fast (~300 ps) phase was smallest [at the beginning of the onset of the autocatalytic effect, i.e., before the third (*) or the fourth (**) strong flash; see Fig. 2 for C8 FA]. All traces are averages of 64 signals recorded upon excitation by weak (~20 μJ/cm^2^) flashes at 355 nm. (**B**) Share of the ~300-ps phase in the TRF signals with added substrates shown in (A), corresponding to the forward electron transfer from the substrate to the photoexcited FAD followed by the quasi-instantaneous CO_2_ cleavage (*13*). The relatively high minimum share of the fast phase in the C8 FA TRF signal (recorded before the third strong flash) indicates that C8 FA itself probably also acts to some extent as a cocatalyst for the decarboxylation of another C8 FA molecule, although not as efficiently as its C7 alkane product.

**Fig. 2.**
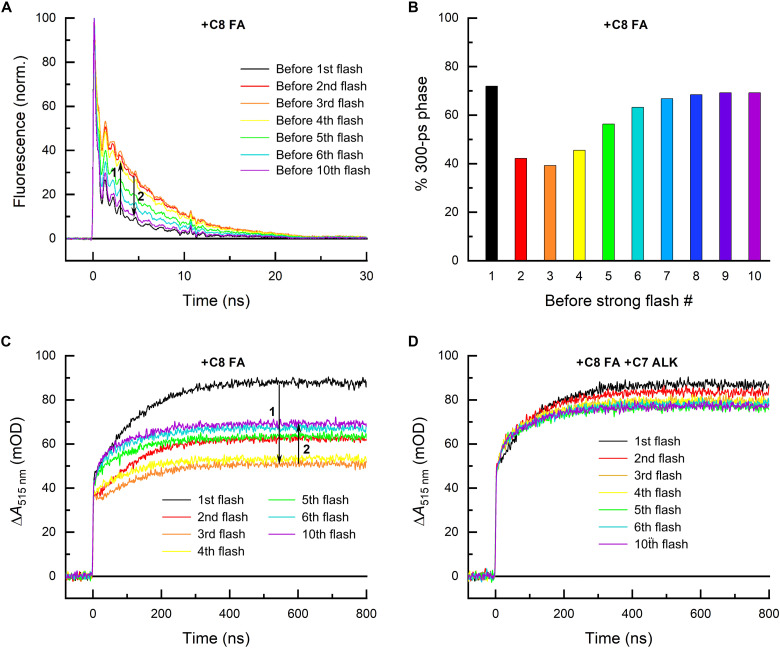
Auto-/cocatalysis of saturated linear C8 FA photodecarboxylation by its C7 *n*-alkane product. (**A**) Normalized TRF signals at 560 nm recorded for ~30 μM *Cv*FAP with added ~300 μM C8 FA prior to 10 strong 470-nm flashes progressively consuming the native C18 FA substrate and the added C8 FA. The first two flashes led to the decrease of the amplitude of the 300-ps phase, but the subsequent flashes led to its gradual recovery (“yo-yo” effect). (**B**) Share of the ~300-ps phase in the TRF signals with added substrates shown in (A), reflecting the quantum yield of photodecarboxylation. The initial flashes consuming most of the native substrate and also small amounts of C8 FA result in a gradual onset of autocatalysis by the formed C7 alkane product. (**C**) Transient absorption changes at 515 nm on the submicrosecond time scale recorded upon the strong 470-nm flashes. The step-like initial increase (reflecting the reduction of FAD to the FAD^∙−^ radical in ~300 ps) is followed by further growth with a time constant of ~100 ns, in line with the back ET and the formation of red-shifted reoxidized flavin (FAD_RS_) (*1*, *13*). The yo-yo effect is also clearly visible in the TAS signals. (**D**) TAS signals at 515 nm recorded for a sample with the same *Cv*FAP and C8 FA concentrations as the one used for experiments shown in (A) and (C) but in the presence of additional ~3 mM C7 *n*-alkane (ALK). With C7 alkane present from the start, the yo-yo effect disappears. mOD, milli Optical Density.

To record TRF and TAS signals corresponding to the complex of *Cv*FAP with the given added substrate, the strongly bound native C18 substrates (*1*, *13*) first had to be consumed (see fig. S1, A and B) by several strong (~10 mJ/cm^2^) laser flashes at 470 nm (near the absorption maximum of FAD in FAP). In line with the previous studies (*1*, *15*), the activity of *Cv*FAP on all studied medium-chain FA substrates (C7 to C12 FAs) as judged by the amplitude ratios in the TRF signals (recorded at 560 nm; see Materials and Methods for reasons why this particular wavelength was chosen) after five (or in some cases less; see Fig. 1 legend for details) strong laser flashes (Fig. 1, A and B) was substantially lower than for longer substrates such as C14, C16, or C18 FAs (Fig. 1, A and B). However, in the case of *n*-octanoic acid (C8 FA) and to a lesser extent for *n*-heptanoic, *n*-nonanoic, and *n*-decanoic acids (C7, C9, and C10 FAs, respectively), further flashes led to a decrease in the slow fluorescence phase (attributed to nonproductive intersystem crossing of the excited flavin) and to an increase in the fast phase reflecting productive forward ET from the substrate to ^1^FAD* leading to decarboxylation (Fig. 2, A and B). After several further laser flashes, the C8 FA TRF signals began to resemble those recorded before consumption of the native (likely unsaturated) C18 FA substrate, or those recorded, e.g., for saturated C16 FA in one of the control experiments (see fig. S2, A and B). This “yo-yo” effect (i.e., an initial decrease in the amplitude of the fast fluorescence phase followed by its recovery upon subsequent flashes) was also observed in the transient absorption signals measured at 515 nm (Fig. 2C), where the initial two flashes led to ever diminishing amounts of FAD_RS_, but this trend was reversed after the third flash and additional flashes led to an ever increasing amplitude of the ~100-ns kinetic phase corresponding to formation of FAD_RS_ (note that FAD_RS_ is one of the products of a successful decarboxylation). Suspecting an autocatalytic effect of the product (*n*-heptane in the case of C8 FA), we added *n*-heptane (C7 alkane) to a fresh sample of *Cv*FAP containing native substrate(s) and C8 FA. Upon addition of C7 alkane, the yo-yo effect observed in the previous experiments disappeared, and immediately after the consumption of the native C18 FA substrate, the signals stabilized at a similar or even slightly higher (due to the C7 alkane excess) level as after 10 flashes in the experiment without the added C7 alkane (Fig. 2D versus Fig. 2C), suggesting that our assumption was correct and that the recovered yield of C8 FA photodecarboxylation after multiple flashes (Fig. 2, A to C) is due to the presence of C7 alkane generated by decarboxylation of C8 FA upon the preceding flashes (note that the observed quantum yield recovery also shows that the protein was not notably damaged in the course of the experiments).

**Fig. 3.**
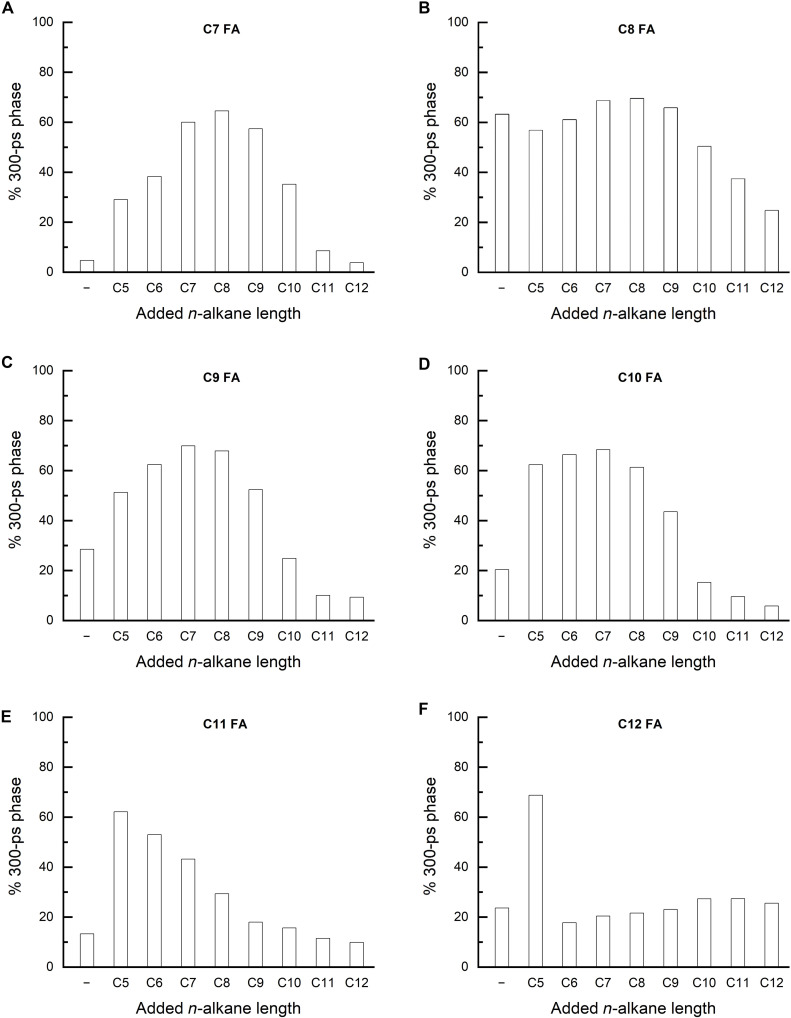
Effect of C5 to C12 *n*-alkane cocatalysts on the photodecarboxylation of saturated linear C7 to C12 FAs by *Cv*FAP. (**A** to **F**) Share of the ~300-ps phase in the TRF signals at 560 nm recorded for ~30 μM *Cv*FAP with added ~300 μM C7 to C12 FAs in the absence (−) and in the presence of ~3 mM C5 to C12 *n*-alkanes as cocatalysts after five strong 470 nm flashes (i.e., after the consumption of the native substrate).

These observations inspired us to perform another set of screening experiments, in which we added C5 to C12 *n*-alkanes to the *Cv*FAP samples containing C7 to C12 FAs. Our results summarized in Fig. 3 (A to F) show that added *n*-alkanes can serve as cocatalysts and enhance the quantum yield of medium-chain FA decarboxylation to ~70%, i.e., to levels as high as those observed for long-chain substrates (Fig. 1).

### Molecular dynamics simulations

**Fig. 4.**
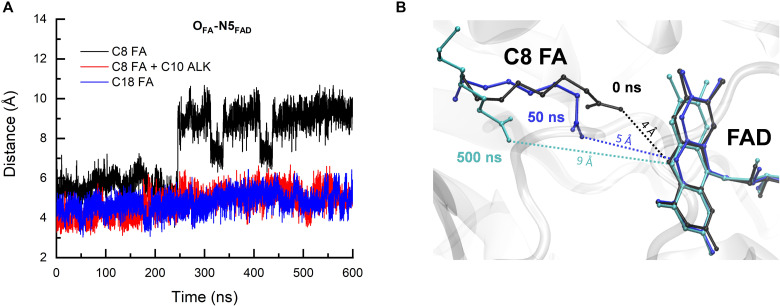
MD simulations. (**A**) Dynamics of the distances between the carboxyl O atoms of FAs and the N5 atom of the FAD isoalloxazine ring during the MD simulations of FAP in complex with C8 FA alone (black trace), C8 FA together with C10 alkane (red trace), and C18 FA (blue trace). (**B**) Snapshots of the active site structure from the MD simulation of FAP with C8 FA alone taken at selected simulation time points. The dotted lines show the evolution of the distance followed in (A). See fig. S3 for more data.

We performed molecular dynamics (MD) simulations with classical force fields to investigate the effects of a cocatalyst in the protein active site (Fig. 4 and figs. S3 and S4). MD simulations lasting 600 ns were performed for FAP with the following molecules in the active site: (i) C8 FA alone, (ii) C8 FA with C10 alkane as a cocatalyst, and (iii) C18 FA (Fig. 4 and fig. S3). Here, the combination of C8 FA and C10 alkane was preferentially chosen as the native substrate present in the high-resolution crystal structure (*13*), which contains 18 carbon atoms. To investigate the effect of a different chain length, MD simulations lasting 400 ns were also performed for C8 FA with C7 alkane (combination of FA and its decarboxylation product exhibiting the most pronounced autocatalytic effect in the experiment; see Fig. 3) and the corresponding C15 FA (fig. S4). Two additional 500-ns simulations were performed for C8 FA alone starting with different conditions for verification (fig. S3).

As shown in Fig. 4A and fig. S3C, with C10 alkane filling the rest of the substrate-binding tunnel closer to the protein surface, C8 FA was able to stay close to the FAD cofactor throughout the MD simulations. Its position was similar to that of C18 FA in the active site observed in the experimental structure (fig. S3C), with an average distance of 4.1 Å between the terminal carbons of C8 FA and C10 alkane. Similar results were obtained in simulations with C8 FA and C7 alkane (fig. S4). However, because of the longer average distance (4.9 Å) between the carbons of the C8 FA tail and the C7 alkane head, C8 FA could transiently move away from FAD and closer to C7 alkane, as shown in fig. S4 (A to C). The position of C7 alkane remains unchanged (fig. S4C), thereby preventing C8 FA from further dissociation and, as a result, C8 FA eventually returns to its original position in the active site after ~50 ns (fig. S4, A and C). In the absence of alkane, however, substantial structural fluctuations of C8 FA were observed (Fig. 4, A and B), regardless of the initial structures, as demonstrated by independent MD simulations (fig. S3A); the C8 FA substrate changed conformations, occupied new positions in the substrate tunnel, and could move as far as ~9 Å away from FAD (Fig. 4, A and B, and fig. S3). During these rearrangements, carboxyl O atoms of the C8 FA still maintained close interaction with N^η^ of the arginine R451 (for most of the simulation time; see fig. S3B), which is a crucial residue for the positioning of the substrate (*13*), and the space near the FAD previously occupied by the carboxyl group of C8 FA was gradually filled by water molecules.

### FA decarboxylation efficiency under continuous light

**Fig. 5.**
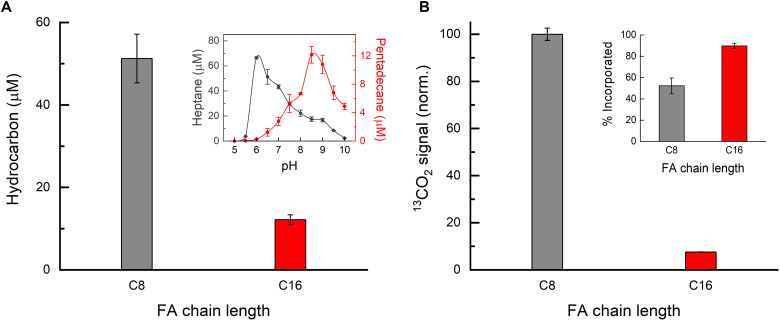
In vitro and in vivo decarboxylation of saturated linear C8 and C16 FAs by *Cv*FAP under continuous light. (**A**) *Cv*FAP activity on C8 and C16 FAs in vitro expressed in terms of concentrations of the corresponding hydrocarbon products obtained at concentrations close to their respective decarboxylation efficiency saturation levels (~25 mM C8 and ~0.5 mM C16 FA; see fig. S5) and at the optimal pH (6 for C8 and 8.5 for C16 FA; see inset) under identical illumination conditions. Inset: pH dependence of *Cv*FAP activity on C8 FA (dark gray curve) and on C16 FA (red curve). (**B**) *Cv*FAP activity on C8 and C16 FAs in vivo (in living *E. coli* bacteria) based on amplitudes of the ^13^CO_2_ signals obtained by gas chromatography–mass spectrometry (GC-MS) analysis and normalized to the C8 signal. Inset: Incorporation of C8 and C16 FAs into the *E. coli* cells after 24 hours of dark incubation at 18°C. The error bars show the SD obtained performing the experiments with three independent samples.

The selective autocatalytic effect observed for C7 to C10 FAs in the spectroscopic experiments described above raised a question about whether this phenomenon also leads to FAP activity enhancement under continuous illumination. To address this question, we compared the in vitro activity (chemical yield of decarboxylation) of purified *Cv*FAP on C16 FA (one of the best FAP substrates described so far) (*1*, *4*) and C8 FA (the substrate showing the most pronounced autocatalytic effect in this study). Given that FAP is an interfacial enzyme (*16*), the saturation of the enzymatic activity for FAs of various chain lengths may occur at very different pH values andsubstrate concentrations, because these parameters strongly influence the structuring of FAs (*17*) and thereby the activity of the enzyme. So far, all FAP activity comparisons on different substrates were done essentially at one pH value and with the same substrate concentration. This approach may introduce a bias by favoring the protodecarboxylation of certain substrates. To eliminate such bias, we decided to determine the pH optimum for photoconversion of the two compared substrates (C8 and C16 FAs). We screened pH values from 5 to 10 (in the universal Teorell-Stenhagen buffer) and found maximum activity at pH 6.0 for C8 and at pH 8.5 for C16 FAs; see inset of Fig. 5A. Using the optimum pH values, we compared the activity of *Cv*FAP at the activity saturating concentration of the individual substrates (25 mM for C8 FA and 0.5 mM for C16 FA; see fig. S5) by quantifying the corresponding products of decarboxylation (C7 and C15 alkanes, respectively). Our results show that *Cv*FAP produced almost four times more C7 alkane from C8 FA than C15 alkane from C16 FA at their respective pH and substrate concentration optima (Fig. 5A).

This result prompted us to evaluate hydrocarbon production in a biotechnological context, where *Cv*FAP would catalyze FA conversion to alkanes inside living cells. *E. coli* bacteria genetically transformed to express *Cv*FAP were therefore grown, and C8 or C16 FAs were added (at the same concentrations, 2 mM) to the culture medium before illuminating the samples and quantifying the hydrocarbon production. To avoid technical biases related to the volatility of the different *n*-alkane products (C7 versus C15), we used FAs that were ^13^C-labeled on the carboxylic group and measured the side product of the reaction, ^13^CO_2_. The ^13^CO_2_ formed by cell cultures contained in sealed flasks upon 1-hour illumination was measured by static headspace [gas chromatography–mass spectrometry (GC-MS) analysis of volatile or semivolatile components in the gas phase above the liquid phase after equilibration in a closed vial; see the “Analysis and quantification of ^13^CO_2_” section for more details). The results show that *Cv*FAP expressing cell cultures produced almost 13 times more ^13^CO_2_ from C8 FA than from C16 FA under otherwise identical experimental conditions (Fig. 5B). The large difference in the efficiency of C8 and C16 FAs decarboxylation is not likely to be due to a problem of penetration of C16 FA into the cells because, before the start of illumination, there was only 10% C16 FAs remaining in the culture medium (see inset of Fig. 5B).

## DISCUSSION

Screening the quantum yield of medium-chain (C7 to C12) FAs photodecarboxylation by *Cv*FAP using time-resolved spectroscopy, we observed an autocatalytic effect, whereby the initially formed *n*-alkane products enhanced the decarboxylation of further FA substrates by mimicking the missing part of the long chain, for which the FA binding site of FAP is adapted. This autocatalytic effect was observed for C7 to C10 FAs but it was most pronounced for the C8 FA substrate. These observations inspired us to test the effects of C5 to C12 *n*-alkanes added as cocatalysts to C7 to C12 FA substrates. Our results show that *n*-alkanes can serve as cocatalysts and decarboxylation quantum yields previously achievable only for long-chain FAs can now be obtained also for medium-chain (C7 to C12) FAs, provided that the right combination of FA and *n*-alkane is chosen. The highest quantum yields (60 to 70%) were obtained when the total number of carbon atoms of the FA substrate and the *n*-alkane cocatalyst was 16 ± 1, in line with the most pronounced autocatalytic effects observed for C8 FA and with C16 FA being one of the best reported substrates for *Cv*FAP (*1*) [note that the ideal length of ~16 total carbon atoms was also suggested by Zhang *et al.* (*15*), who attempted to enhance *Cv*FAP enzymatic activity under continuous light on short (C1 to C6) substrates by adding C7 to C17 *n*-alkanes to crude cell extracts from *E. coli* expressing *Cv*FAP].

The outcomes of our MD simulations suggest that the auto- and/or cocatalytic effect of the *n*-alkanes can be attributed to a steric hindrance that favors the positioning of the medium-chain FAs close enough to the FAD cofactor so that the initial forward electron transfer from the substrate to FAD can occur upon photoexcitation of the latter. The simulations also establish that the conformation of C8 FA and C10 or C7 alkanes closely resembles that observed in the MD simulations with the corresponding long-chain FAs (C18 or C15, respectively).

Our in vitro assay demonstrates that the autocatalytic effect (observed in the spectroscopic experiments and rationalized by MD simulations) together with the optimum pH and substrate concentration can considerably enhance the photoconversion of a medium-chain FA (C8 FA) by *Cv*FAP under continuous illumination. The chemical yield of C8 FA decarboxylation exceeding four times that of C16 FA is the best in vitro performance of wild-type *Cv*FAP on a medium-chain substrate reported so far. The difference in the optimum pH obtained for C8 and C16 FAs (6.0 and 8.5, respectively) is consistent with experimental data on the p*K*_a_ of C8 to C16 saturated FA salts organized in films (*18*), as well as quantum chemistry calculations, which show that the lengthening of an FA carbon chain by one methylene group leads to an increase of 0.43 units in the surface p*K*_a_ at the air-water interface (*19*).

Last, our in vivo assay (photodecarboxylation of 1-^13^C–labeled C8 and C16 FAs inside the living *E. coli* bacteria) shows that the chemical yield of medium-chain *n*-alkanes by FAP under continuous irradiation can greatly exceed that of long-chain ones, by a factor of ~13 for the case of C7 and C15 *n*-alkanes. The autocatalytic effect certainly contributes to the efficient decarboxylation of medium-chain FAs, but autocatalysis alone is not sufficient to explain the magnitude of this difference. Given that the permeability of the cell membranes seems to be higher for long substrates (such as C16 FA) and that the kinetics of the initial photochemical steps are also similar (for both C8 and C16 FAs), we suggest that the high chemical yield of medium-chain (C8) FA decarboxylation observed both in vitro and in vivo is most likely due to an acceleration of the “dark” steps of the photocycle, namely, the exchange of the product for a new substrate. Faster replacement of the product with a new substrate (in the case of medium-chain FAs) may also have a positive impact on the (photo)stability of the enzyme (*20*).

In conclusion, the present work unveils an autocatalytic effect of C7 to C10 FAs decarboxylation products and documents the cocatalytic effects of C5 to C12 *n*-alkanes on the decarboxylation of medium-chain (C7 to C12) FAs by *Cv*FAP. It further reveals an unexpectedly high in vivo activity of *Cv*FAP on C8 FA acid, which is over 10-fold higher than that on C16 FA, the best FAP substrate identified before our current study. These results should stimulate applied research on the use of *Cv*FAP to convert medium-chain substrates and guide future studies aiming at improving *Cv*FAP for high-yield production of gasoline-like hydrocarbons.

## MATERIALS AND METHODS

### FAP expression

The coding sequence corresponding to *C. variabilis* NC64A FAP was cloned into a pLIC07 plasmid (pLIC07 is a pET-28–based expression vector containing downstream FAP gene, a histidine-tagged thioredoxin). The version of *Cv*FAP used here is FAPv2, which corresponds to the full-length protein truncated by the first 75 amino acids at the N terminus (*13*, *16*). The plasmid pLIC07FAP was then transformed into a BL21 *E. coli* expression strain harboring the pRIL plasmid that allows the synthesis of the rare transfer RNAs that are not naturally produced in *E. coli* cells. For protein production, after overnight preculture in LB media at 37°C and 180 rpm agitation, the strain was grown in terrific broth (TB) medium supplemented with 0.5% (v/v) glycerol at 37°C, 180 rpm, to an absorbance of 1 before induction with 500 μM isopropyl-β-d-thiogalactopyranoside (IPTG). The temperature was then lowered to 18°C and the cultures were incubated for 24 hours. The cells were harvested by centrifugation at 8°C, 5000 rpm for 30 min, and the pellets were stored in a freezer at −80°C.

### FAP purification and quantification

The first purification step consisted in thawing the frozen pellets. The cells were resuspended in a buffer containing 300 mM NaCl, 50 mM tris-HCl (pH 8.0), 10 mM imidazole, and 5% (w/v) glycerol. To allow cell lysis, lysozyme was added at a final concentration of 0.25 mg ml^−1^. DNA digestion was done by adding deoxyribonuclease and MgSO_4_ (final concentrations of 10 μg ml^−1^ and 20 mM, respectively). Antiprotease tablets (Sigma-Aldrich, S8820-20 TAB) were also dissolved in the lysis buffer at the rate of one tablet per 100 ml of buffer (as a rule of thumb, for initially 8 liters of cultures, 500 ml of lysis buffer was used). After incubation for 1 hour at room temperature, the cells were treated by sonication to improve bacterial lysis. Sonication parameters were as follows: 20 kHz, 4 cycles of 45 s with 15-s pause and agitation to avoid overheating. Soluble proteins were recovered after centrifugation at 11,000*g* for 30 min at 4°C. FAP was purified by affinity chromatography using a nickel column [elution buffer: 5% (w/v) glycerol, 300 mM NaCl, 50 mM tris-HCl (pH 8.0), and 250 mM imidazole]. To get rid of the histidine tag in fusion with thioredoxin, the eluate was then digested for at least 1 hour at room temperature using tobacco etch virus (TEV) protease (1 mg of TEV for 20 mg of protein to be digested). The digestate was dialyzed (membrane reference: Spectra Por/Standard RC tubing, molecular weight cut-off: 12 to 14 kDa) overnight against 300 mM NaCl, 50 mM tris (pH 8.0), 10 mM imidazole, and 5% (w/v) glycerol. The FAP protein was then separated from the previously cut histidine tag by a second affinity chromatography using a nickel column. A final purification step by gel filtration using Superdex 200 26/600 mm (GE HealthCare) column was necessary to separate any aggregates from the soluble protein. The buffer used for this step contained 150 mM NaCl, 10 mM tris (pH 8.0), and 5% (w/v) glycerol. In general, after all the purification steps, we obtained a protein of >95% purity. The purified protein was quantified based on the absorbance at 280 nm (*13*). To determine the amount of active protein (i.e., protein containing FAD), the FAD concentration was estimated by dividing the absorbance at the maximum of the oxidized FAD band in the blue (467 nm) (*13*), by the typical molar extinction coefficient of FAD in flavoproteins: 11,300 M^−1^ cm^−1^ (*21*). Typically, 70 to 85% FAP proteins contained bound FAD. After purification, the protein was concentrated using ultracentrifuge filters 50-kDa Amicon up to 20 mg ml^−1^ flash-frozen in liquid nitrogen and stored at −80°C.

### Time-resolved fluorescence

Fluorescence kinetics were monitored on a setup described previously (*1*, *13*), using “weak” flashes from a neodymium-doped yttrium aluminum garnet (Nd:YAG) laser as the excitation light source (Continuum Leopard SS-10; pulse duration of 100 ps, 355 nm, energy attenuated to ∼20 μJ/cm^2^, and repetition rate 2 Hz). “Strong” (~10 mJ) flashes at 470 nm of ∼5-ns pulse duration used to consume the native and/or the added substrates were delivered using a Nd:YAG–pumped optical parametric oscillator (Brillant B/Rainbow, Quantel, France). The detection system consisted of a Hamamatsu microchannel plate photomultiplier tube R2566U-11P (under 3.6 kV) connected to a digital oscilloscope (Tektronix MSO64; bandwidth limit set to 6 GHz). The samples were contained in a 2-mm by 2-mm by 10-mm cell with self-masking solid black walls and four clear windows (Starna). Excitation pulses entered through the 2-mm by 10-mm window, and fluorescence was detected in front of the 2-mm by 2-mm window. An OG-530 Schott orange glass optical filter and a 560-nm interference filter (with a 10-nm bandwidth) were placed between the sample and the detector. The fluorescence emission spectrum of ^1^FAD* spreads between 480 and 620 nm, with maxima typically around 530 nm. The wavelength of 560 nm is still well within the ^1^FAD* emission band (with ~80% of the maximum intensity) but far enough from 532 nm, the second harmonic of the used Nd:YAG laser, traces of which could have also been present in our 355-nm pulses. Hence, the wavelength of 560 nm was chosen to avoid possible contaminations of the fluorescence signals by photons from the excitation source.

Fluorescence traces are averages of 64 signals. A total of ~30 μM active (FAD-containing) *Cv*FAP protein was used in the presence (or absence) of ~300 μM substrates, ~3 mM *n*-alkane cocatalysts, and 5% ethanol [substrates and alkanes were dissolved in ethanol and added to the aqueous solution of *Cv*FAP before each experiment; for consistency, ethanol was added (to the final concentration of 5%) also to the samples without added substrates and alkanes].

Normalized TRF signals were fitted using the Levenberg-Marquardt least square optimization algorithm in Origin 2020 (by OriginLab). The signals with two pronounced phases (~300 ps and ~5 ns) could be reasonably fitted by a biexponential function *y* (*t*) = *A*_1_ × e^–*t*/τ1^ + *A*_2_ × e^–*t*/τ2^, where *A*_1_ and *A*_2_ are the amplitudes of the fast and of the slow phase, respectively (their sum should be equal to 1 because the signals are normalized), and τ_1_ and τ_2_ are their respective time constants; *t* stands for time (in seconds). However, when the fast phase was small relative to the slow one, the inherent oscillatory artifact at the beginning of our signals [natural response of the photomultiplier to a rapid (~100 ps) change in light intensity] made a correct fit of the fast phase impossible [see figure 5A in (*1*) for the instrument response signal and SI text ibid for more details]. Therefore, we fitted only the slow phase, starting the fit at *t* = 1 ns, i.e., after the end of the ~300-ps process (more than three times its time constant), using a simple (monoexponential) function *y* (*t*) = *A*_2_ × e^–*t*/τ2^. *A*_1_ was subsequently calculated using *A*_1_ = 1 − *A*_2_. We verified on several signals with pronounced fast phases that this method yielded the same results for *A*_1_ and *A*_2_ as the biexponential fit of the complete curves fitted from *t* = 0 ns (with an error of max. 2%). For simplicity, the amplitudes are recalculated to percentage in the figures (*A*_1_ + *A*_2_ = 1 = 100%).

### Transient absorption spectroscopy

Transient absorption kinetics was also recorded on a setup described previously (*1*, *13*). The FAD cofactor in FAP was excited at 470 nm by laser flashes identical to the strong flashes in the TRF experiment (i.e., ∼5-ns pulse duration and energy in the order of 10 mJ/cm^2^ delivered using a Nd:YAG–pumped optical parametric oscillator Brillant B/Rainbow, Quantel, France). The monitoring light at 515 nm was provided by the DPSS laser Cobolt Fandango (150 mW). The sample cell was identical to that used in the TRF experiments (2 mm by 2 mm by 10 mm). The monitoring light was attenuated by neutral density filters and mechanically chopped to produce a rectangular pulse of ∼140-μs duration and an energy in the order of 1 μJ at the entrance of the cell (2-mm by 2-mm window), thus avoiding significant actinic effects. This pulse was synchronized with the excitation laser flash entering the sample through the 2-mm by 10-mm window. The signals were recorded using an Alphalas UPD-500-UP photodiode (rise time < 500 ps; sensitive area of 0.5 mm^2^) connected via a Femto HCA electronic signal amplifier (DC-325 MHz, 28 dB) to the Tektronix MSO64 digital oscilloscope with the bandwidth limit set to 200 MHz. The samples used in TAS experiments were prepared in the same way as those used in the TRF experiments (see above). For consistency with the TRF experiments (to allow the substrates and cocatalysts the same time to reorganize after the strong flashes), the excitation flashes were separated by delays of ∼2 min (about the time needed for one fluorescence measurement).

### MD simulations

The structure of *Cv*FAP was taken from the Protein Data Bank (PDB entry: 6ZH7; resolution: 2.0 Å). In this structure, two molecules of C18 FA (presumably unsaturated) are present; the one located at the surface of the enzyme close to the entrance to the tunnel leading to the binding site was removed before simulations. The system with saturated linear C8 FA and C7 alkane was constructed by removing the “C3-tail” and the covalent bond from C18 FA in the crystal structure and by adding two hydrogens to complete the methyl groups of C8 FA and C7 alkane. To construct the system with C8 FA and C10 alkane, a similar approach was used, but no carbon atoms were removed. Three simulations of *Cv*FAP with C8 FA alone were performed with the initial structure taken from the equilibrated trajectory of the simulation with C8 FA and C10 alkane (with C10 alkane removed). The results of the latter were used in discussion, as the results of all three simulations were very similar.

The CHARMM36 force field was used for the protein residues (*22*), the modified version of the TIP3P model for water molecules (*23*), and a recently developed force field for flavins for the FAD cofactor (*24*). The parameters for C8, C15, and C18 FAs and for C7 and C10 alkanes were taken from the CHARMM General Force Field (CGenFF, version 2.2.0) (*25*). The protonation states of all titratable residues were assigned based on a PROPKA 3.1 analysis (*26*) and verified by ideal stereochemistry, taking into account steric effects and potential hydrogen bonding interactions.

The systems were then centered in a cubic box of aqueous solvent with an appropriate size, at least 12 Å away from each of the box edges; thus, the final systems in addition to the *Cv*FAP protein, FAD, and substrates contained around 30,000 water molecules. MD simulations were performed using the NAMD program (version 2.13) (*27*). Periodic boundary conditions were assumed with long-range electrostatic interactions computed using the particle mesh Ewald method (*28*), and an appropriate number of potassium counterions was included to neutralize the net charge of the systems. The integration time step was set to 2 fs. After energy minimization, the system was equilibrated first in a constant temperature (NVT) ensemble for 50 ps, followed by a 500-ps simulation in the isothermal-isobaric (NPT) ensemble, at 295 K and 1.0-atm (101.325 kPa) pressure. The Berendsen thermostat and barostat were used, with a relaxation time of 500 fs and four time steps between position rescalings for constant pressure simulations (*29*). The production runs were then performed, and coordinates of the systems were collected every 100 ps.

### In vitro photoenzymatic production of *n*-alkanes

In vitro assay was performed using the purified *Cv*FAP. Teorell-Stenhagen buffer (33 mM citric acid monohydrate, 33 mM phosphoric acid and 16.7 mM boric acid) was used for the enzymatic reaction. The buffer was mixed with pure FA solution before adjusting the pH to the desired value using NaOH (10 M) or HCl (6 N) solutions. The protein solution was then added in the dark from a 1000-fold concentrated stock to obtain 70 nM protein in 5 ml of final reaction volume (contained in 10-ml vials, i.e., 5-ml liquid and 5-ml gas phase). The samples were then exposed to light at 360 μmoles photons/m^2^/s of blue light-emitting diode (LED) light (450 nm; full width at half maximum: 20 nm) at 25°C for 20 min to induce photodecarboxylation.

### Analysis and quantification of C7 alkane

After C7 alkane production under light, the reaction was stopped by enzyme denaturation at 100°C for 10 min. The samples were then cooled at room temperature for 30 min to recondense all volatile alkanes. Then, the samples were reheated to 40°C and kept at this temperature for 5 min. One milliliter of gas phase was collected with a syringe heated at 80°C and injected into a GC-MS/FID (flame ionization detector) [Agilent Technologies, 5977B series mass selective detector (MSD)] for analysis using helium as the carrier gas. The analysis parameters were as follows: oven initial temperature, 50°C for 1 min; ramp, 20°C/min to 260°C for 5 min (column reference: 19091P-Q04PT; PH-PLOT/Q + RT; 30 m by 0.320 mm, 20 μm). The absolute amount of C7 alkane was calculated from an external standard curve obtained using pure C7 alkane solutions. The curves were processed under the same conditions as those used for the test reactions.

### Analysis and quantification of C15 alkane

After C15 alkane production under light, 100 μl of NaOH (10 M) was added before heating at 100°C during 10 min. Four milliliters of hexane and 10 μg of *n*-hexadecane (C16 alkane, internal standard for C15 alkane quantification) were added. The samples were then vortexed and centrifuged (3000 rpm, 5 min). The organic phase containing the extracted C15 alkane was collected, and 1 μl was injected in GC-MS/FID for analysis according to the following program: oven initial temperature, 60°C for 1 min; ramp, 20°C/min to 150°C; 10°C/min to 260°C for 2 min. Column reference is as follows: OPTIMA WAXplus, 30 m by 0.25 mm, 0.25 μm. The absolute amount of C15 alkane was calculated based on the internal standard added before the sample treatment.

### In vivo photoenzymatic production of *n*-alkanes

The production of *n*-alkanes was performed using the *E. coli* BL21 strain containing pLIC07FAPv2 plasmid. A preculture was grown overnight in LB medium at 37°C, 180 rpm. For protein production, the strain was cultured in TB medium supplemented with 0.5% (w/v) glycerol at 37°C, 180 rpm, to an absorbance of 1 before induction with 500 μM IPTG and addition of 1-^13^C–labeled FA substrates at 2 mM (the concentrated stock solution of C8 and C16 FAs was previously prepared in ethanol). The temperature was then lowered to 18°C, and the cultures were incubated in the dark for 24 hours. Five milliliters of each culture was then put in sealed 10-ml airtight vials and illuminated at 360 μmol photons/m^2^/s of blue LED light (450 nm) at 25°C for 1 hour to induce photodecarboxylation.

### Analysis and quantification of ^13^CO_2_

For ^13^CO_2_ analysis after the illumination, the reaction was stopped by heating the samples to 100°C for 10 min (denaturing the enzymes and lysing the cells to release all trapped volatiles). After cooling the samples, hydrochloric acid was added to the vials to lower the pH to 1 and convert all dissolved bicarbonate to CO_2_. Then, the samples were reheated to 40°C and kept at this temperature for 5 min. One milliliter of gas was collected and injected into a GC-MS/FID (Agilent Technologies, 5977B series MSD) for analysis. The analysis parameters were as follows: oven initial temperature, 50°C for 1 min; ramp: 20°C/min to 260°C for 5 min (column reference: 19091P-Q04PT, PH-PLOT/Q + RT; 30 m by 0.320 mm, 20 μm) using helium as the carrier gas.

### ^13^C-labeled substrate quantification

To quantify the amount of the remaining substrates that were not incorporated into the cells after 24 hours of culture incubation, 1 ml of each was centrifuged. Then, 10 μg of saturated C17 FA methyl ester (standard) was added to 50 μl of the supernatant (note that the cells were also washed to dissolve any remaining FAs at the membrane surface). To perform the transmethylation of the samples, 2 ml of sulfuric acid/methanol (5/100; v/v) were added before heating at 85°C for 90 min. The samples were then cooled to room temperature, and 2 ml of NaCl (0.9%) and 500 μl of hexane were added successively. To extract the transmethylated FAs, the samples were shaken for 10 min and centrifuged at 3000 rpm for 5 min. One microliter of the hexane phase was injected into the GC-MS/FID (Agilent Technologies, 5977B series MSD) using helium as the carrier gas. The analysis parameters were as follows: oven initial temperature, 20°C for 1 min; ramp, 20°C/min to 300°C for 1 min using helium as the gas carrier. Column reference is as follows: 190915-433UI, HP-5MS UI, 30 m by 0.250 mm, 0.25 μm. The results shown in Fig. 5B are the averages of values obtained in three independent samples normalized by the absorbance. The absolute amount of C8 and C16 FA methyl esters was calculated on the basis of the internal standard (i.e. C17 FA methyl ester) added before the sample treatment.

## Notes

View/request a protocol for this paper from *Bio-protocol*.

## References

[1] D. Sorigué, B. Légeret, S. Cuiné, S. Blangy, S. Moulin, E. Billon, P. Richaud, S. Brugière, Y. Couté, D. Nurizzo, P. Müller, K. Brettel, D. Pignol, P. Arnoux, Y. Li-Beisson, G. Peltier, F. Beisson, An algal photoenzyme converts fatty acids to hydrocarbons. Science 357, 903–907 (2017). 28860382 10.1126/science.aan6349

[2] D. Ramírez-Gamboa, A. L. Díaz-Zamorano, E. R. Meléndez-Sánchez, H. Reyes-Pardo, K. R. Villaseñor-Zepeda, M. E. López-Arellanes, J. E. Sosa-Hernández, K. G. Coronado-Apodaca, A. Gámez-Méndez, S. Afewerki, H. M. N. Iqbal, R. Parra-Saldivar, M. Martínez-Ruiz, Photolyase production and current applications: A review. Molecules 27, 5998 (2022). 36144740 10.3390/molecules27185998 PMC9505440

[3] D. J. Heyes, S. Zhang, A. Taylor, L. O. Johannissen, S. J. O. Hardman, S. Hay, N. S. Scrutton, Photocatalysis as the ‘master switch’ of photomorphogenesis in early plant development. Nat. Plants 7, 268–276 (2021). 33686224 10.1038/s41477-021-00866-5

[4] M. M. E. Huijbers, W. Zhang, F. Tonin, F. Hollmann, Light-driven enzymatic decarboxylation of fatty acids. Angew. Chem. Int. Ed. 57, 13648–13651 (2018). 10.1002/anie.201807119 PMC6197046 30106504

[5] I. S. Yunus, J. Wichmann, R. Wördenweber, K. J. Lauersen, O. Kruse, P. R. Jones, Synthetic metabolic pathways for photobiological conversion of CO_2_ into hydrocarbon fuel. Metab. Eng. 49, 201–211 (2018). 30144559 10.1016/j.ymben.2018.08.008

[6] S. Moulin, B. Légeret, S. Blangy, D. Sorigué, A. Burlacot, P. Auroy, Y. Li-Beisson, G. Peltier, F. Beisson, Continuous photoproduction of hydrocarbon drop-in fuel by microbial cell factories. Sci. Rep. 9, 13713 (2019). 31548626 10.1038/s41598-019-50261-6 PMC6757031

[7] S. Bruder, E. J. Moldenhauer, R. D. Lemke, R. Ledesma-Amaro, J. Kabisch, Drop-in biofuel production using fatty acid photodecarboxylase from *Chlorella variabilis* in the oleaginous yeast *Yarrowia lipolytica*. Biotechnol. Biofuels 12, 202 (2019). 31462926 10.1186/s13068-019-1542-4 PMC6708191

[8] J. Xu, J. Fan, Y. Lou, W. Xu, Z. Wang, D. Li, H. Zhou, X. Lin, Q. Wu, Light-driven decarboxylative deuteration enabled by a divergently engineered photodecarboxylase. Nat. Commun. 12, 3983 (2021). 34172745 10.1038/s41467-021-24259-6 PMC8233396

[9] J. Xu, Y. Hu, J. Fan, M. Arkin, D. Li, Y. Peng, W. Xu, X. Lin, Q. Wu, Light-driven kinetic resolution of α-functionalized carboxylic acids enabled by an engineered fatty acid photodecarboxylase. Angew. Chem. Int. Ed. 58, 8474–8478 (2019). 10.1002/anie.201903165 31033108

[10] W. Zhang, J. H. Lee, S. H. H. Younes, F. Tonin, P. L. Hagedoorn, H. Pichler, Y. Baeg, J. B. Park, R. Kourist, F. Hollmann, Photobiocatalytic synthesis of chiral secondary fatty alcohols from renewable unsaturated fatty acids. Nat. Commun. 11, 2258 (2020). 32382158 10.1038/s41467-020-16099-7 PMC7206127

[11] H. J. Cha, S. Y. Hwang, D. S. Lee, A. R. Kumar, Y. U. Kwon, M. Voß, E. Schuiten, U. T. Bornscheuer, F. Hollmann, D. K. Oh, J. B. Park, Whole-cell photoenzymatic cascades to synthesize long-chain aliphatic amines and esters from renewable fatty acids. Angew. Chem. Int. Ed. 59, 7024–7028 (2020). 10.1002/anie.201915108 31957098

[12] D. Sorigué, B. Légeret, S. Cuiné, P. Morales, B. Mirabella, G. Guédeney, Y. Li-Beisson, R. Jetter, G. Peltier, F. Beisson, Microalgae synthesize hydrocarbons from long-chain fatty acids via a light-dependent pathway. Plant Physiol. 171, 2393–2405 (2016). 27288359 10.1104/pp.16.00462 PMC4972275

[13] D. Sorigué, K. Hadjidemetriou, S. Blangy, G. Gotthard, A. Bonvalet, N. Coquelle, P. Samire, A. Aleksandrov, L. Antonucci, A. Benachir, S. Boutet, M. Byrdin, M. Cammarata, S. Carbajo, S. Cuiné, R. B. Doak, L. Foucar, A. Gorel, M. Grünbein, E. Hartmann, R. Hienerwadel, M. Hilpert, M. Kloos, T. J. Lane, B. Légeret, P. Legrand, Y. Li-Beisson, S. L. Y. Moulin, D. Nurizzo, G. Peltier, G. Schirò, R. L. Shoeman, M. Sliwa, X. Solinas, B. Zhuang, T. R. M. Barends, J.-P. Colletier, M. Joffre, A. Royant, C. Berthomieu, M. Weik, T. Domratcheva, K. Brettel, M. H. Vos, I. Schlichting, P. Arnoux, P. Müller, F. Beisson, Mechanism and dynamics of fatty acid photodecarboxylase. Science 372, eabd5687 (2021). 33833098 10.1126/science.abd5687

[14] S. L. Y. Moulin, A. Beyly-Adriano, S. Cuiné, S. Blangy, B. Légeret, M. Floriani, A. Burlacot, D. Sorigué, P. P. Samire, Y. Li-Beisson, G. Peltier, F. Beisson, Fatty acid photodecarboxylase is an ancient photoenzyme that forms hydrocarbons in the thylakoids of algae. Plant Physiol. 186, 1455–1472 (2021). 33856460 10.1093/plphys/kiab168 PMC8260138

[15] W. Zhang, M. Ma, M. M. E. Huijbers, G. A. Filonenko, E. A. Pidko, M. van Schie, S. de Boer, B. O. Burek, J. Z. Bloh, W. J. H. van Berkel, W. A. Smith, F. Hollmann, Hydrocarbon synthesis via photoenzymatic decarboxylation of carboxylic acids. J. Am. Chem. Soc. 141, 3116–3120 (2019). 30673222 10.1021/jacs.8b12282 PMC6385076

[16] C. Aselmeyer, B. Légeret, A. Bénarouche, D. Sorigué, G. Parsiegla, F. Beisson, F. Carrière, Fatty acid photodecarboxylase is an interfacial enzyme that binds to lipid-water interfaces to access its insoluble substrate. Biochemistry 60, 3200–3212 (2021). 34633183 10.1021/acs.biochem.1c00317

[17] D. P. Cistola, J. A. Hamilton, D. Jackson, D. M. Small, Ionization and phase behavior of fatty acids in water: Application of the Gibbs phase rule. Biochemistry 27, 1881–1888 (1988). 3378036 10.1021/bi00406a013

[18] J. R. Kanicky, A. F. Poniatowski, N. R. Mehta, D. O. Shah, Cooperativity among molecules at interfaces in relation to various technological processes: Effect of chain length on the p*K*_a_ of fatty acid salt solutions. Langmuir 16, 172–177 (2000).

[19] Y. B. Vysotsky, E. S. Kartashynska, D. Vollhardt, V. B. Fainerman, Surface p*K*a of saturated carboxylic acids at the air/water interface: A quantum chemical approach. J. Phys. Chem. C 124, 13809–13818 (2020).

[20] Y. Wu, C. E. Paul, F. Hollmann, Stabilisation of the fatty acid decarboxylase from *Chlorella variabilis* by caprylic acid. Chembiochem 22, 2420–2423 (2021). 34002919 10.1002/cbic.202100182 PMC8362199

[21] P. Macheroux, UV-visible spectroscopy as a tool to study flavoproteins. Meth. Mol. Biol. 131, 1–7 (1999). 10.1385/1-59259-266-X:1 10494538

[22] J. Huang, A. D. MacKerell Jr., CHARMM36 all-atom additive protein force field: Validation based on comparison to NMR data. J. Comput. Chem. 34, 2135–2145 (2013). 23832629 10.1002/jcc.23354 PMC3800559

[23] W. L. Jorgensen, J. Chandrasekhar, J. D. Madura, R. W. Impey, M. L. Klein, Comparison of simple potential functions for simulating liquid water. J. Chem. Phys. 79, 926–935 (1983).

[24] A. Aleksandrov, A molecular mechanics model for flavins. J. Comput. Chem. 40, 2834–2842 (2019). 31471978 10.1002/jcc.26061

[25] K. Vanommeslaeghe, E. Hatcher, C. Acharya, S. Kundu, S. Zhong, J. Shim, E. Darian, O. Guvench, P. Lopes, I. Vorobyov, A. D. Mackerell Jr., CHARMM general force field: A force field for drug-like molecules compatible with the CHARMM all-atom additive biological force fields. J. Comput. Chem. 31, 671–690 (2010). 19575467 10.1002/jcc.21367 PMC2888302

[26] M. H. Olsson, C. R. Søndergaard, M. Rostkowski, J. H. Jensen, PROPKA3: Consistent treatment of internal and surface residues in empirical p*K*_a_ predictions. J. Chem. Theory Comput. 7, 525–537 (2011). 26596171 10.1021/ct100578z

[27] J. C. Phillips, R. Braun, W. Wang, J. Gumbart, E. Tajkhorshid, E. Villa, C. Chipot, R. D. Skeel, L. Kalé, K. Schulten, Scalable molecular dynamics with NAMD. J. Comput. Chem. 26, 1781–1802 (2005). 16222654 10.1002/jcc.20289 PMC2486339

[28] T. Darden, D. York, L. Pedersen, Particle mesh Ewald: An *N*⋅log(N) method for Ewald sums in large systems. J. Chem. Phys. 98, 10089–10092 (1993).

[29] H. J. C. Berendsen, J. P. M. Postma, W. F. V. Gunsteren, A. DiNola, J. R. Haak, Molecular dynamics with coupling to an external bath. J. Chem. Phys. 81, 3684–3690 (1984).

