# Supplementary material for: Autocatalytic effect boosts the production of medium-chain hydrocarbons by fatty acid photodecarboxylase
Source: Sci Adv. 2023 Mar 31;9(13):eadg3881. doi: 10.1126/sciadv.adg3881 (PMC10065435; doi:10.1126/sciadv.adg3881)
Supplement: Supplementary file 1 — Figs. S1 to S5 [file sciadv.adg3881_sm.pdf]

Science Advances
AAAS

Supplementary Materials for

Autocatalytic effect boosts the production of medium-chain hydrocarbons by fatty acid photodecarboxylase

Poutoum P. Samire *et al.*

Corresponding author: Pavel Müller, pavel.muller@i2bc.paris-saclay.fr; Frédéric Beisson, frederic.beisson@cea.fr; Alexey Aleksandrov, alexey.aleksandrov@polytechnique.edu

Sci. Adv. **9**, eadg3881 (2023)
DOI: 10.1126/sciadv.adg3881

This PDF file includes:

Figs. S1 to S5

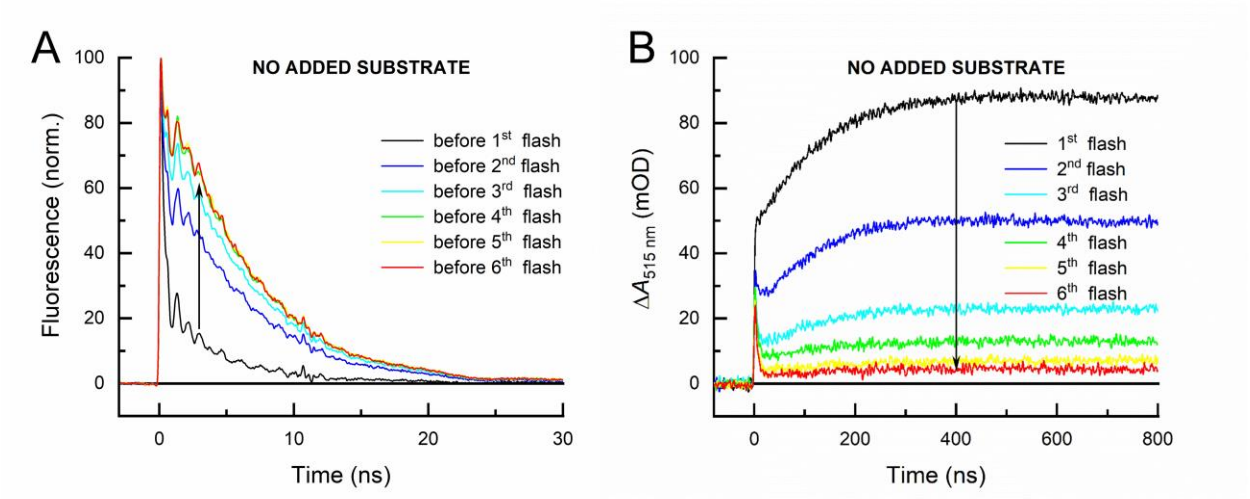

Fig. S1. TRF and TAS signals of *Cv*FAP with the native C18 substrate. (**A**) Normalized TRF signals at 560 nm recorded for ~30 µM *Cv*FAP without an added substrate, *i.e.*, containing maximum two native C18 FAs per enzyme (likely 1 to 1.5 FAs per FAP) prior to six strong (~10 mJ/cm^2) 470 nm flashes progressively consuming the native substrate(s). (**B**) Transient absorption changes at 515 nm on the sub-microsecond timescale recorded for ~30 µM *Cv*FAP without added substrate upon the individual strong excitation flashes. Note that the consumption of the native substrate in the TRF experiment is somewhat accelerated compared to the TAS experiment. This is because some substrate is also consumed upon the 64 weak (~20 µJ/cm^2) flashes at 355 nm used to obtain the fluorescence signals (between the strong 470 nm flashes).

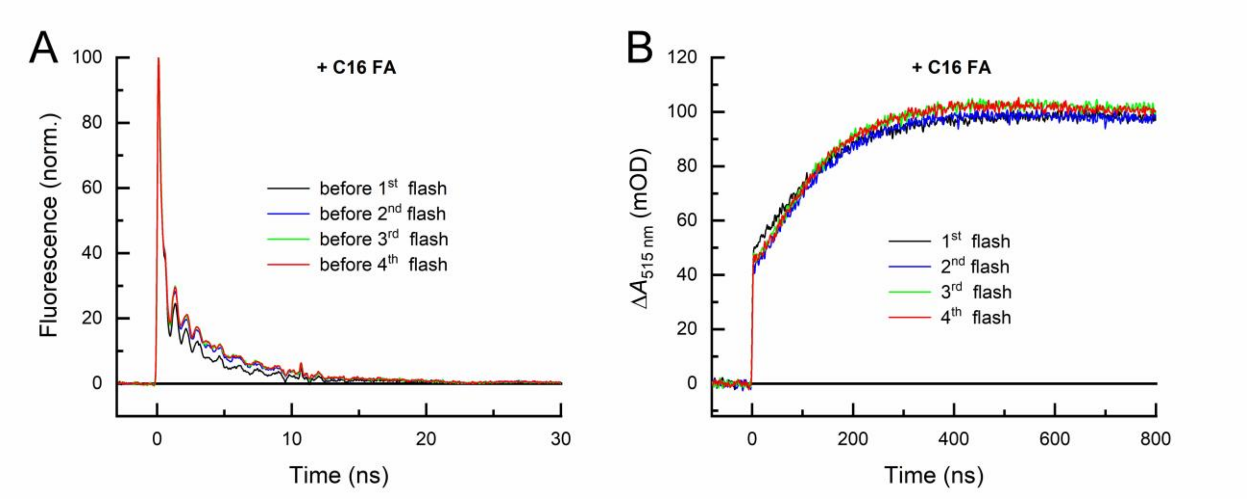

Fig. S2. TRF and TAS signals of *Cv*FAP with added C16 substrate. (**A**) Normalized TRF signals at 560 nm recorded for ~30 μM *Cv*FAP in the presence of ~300 μM C16 FA prior to four strong (~10 mJ/cm^2) 470 nm flashes consuming the native substrate. The share of the ~300 ps phase and hence the yield of C16 decarboxylation appear to be only slightly lower than for the native substrate. (**B**) Transient absorption changes at 515 nm on the sub-microsecond timescale recorded for ~30 μM *Cv*FAP with ~300 μM C16 FA upon the strong 470 nm flashes. Note that although the TRF signals indicate a slightly lower yield of forward ET (consistent with the slightly lower initial stepwise growth of the TAS signal reflecting the $FAD^{\bullet-}$ radical formation), the amplitude of the subsequent ~100 ns growth phase (reflecting FAD_{RS} formation) is larger for C16 than for the native substrate, which is indicative of the dependence of the magnitude of the FAD_{RS} spectral shift on the nature (saturation and/or length) of the substrate/product.

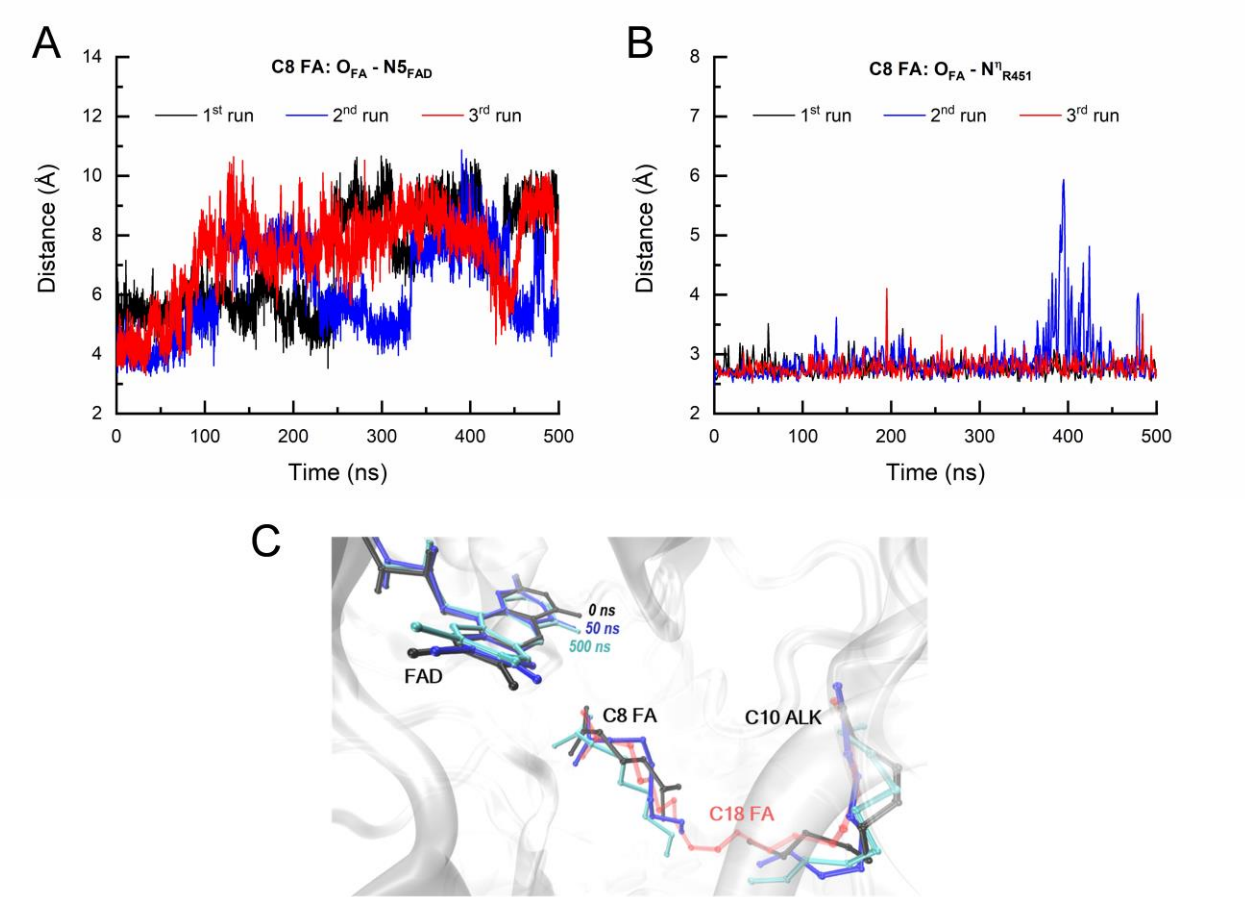

Fig. S3. MD Simulations of *Cv*FAP containing C8 FA alone, C8 FA + C10 alkane, or C18 FA. Dynamics of the distances between: (**A**) the carboxyl O atoms of C8 FA and the N5 atom of the FAD isoalloxazine ring, and (**B**) the carboxyl O atoms of C8 FA and the N^{η} atom of the arginine R451, during the MD simulations of FAP in complex with C8 FA alone (without a co-catalyst) in three independent runs starting with different initial conditions. The 1st run is identical to the one shown in Fig. 4A of the main text (black trace). (**C**) Snapshots from the simulation of C8 FA + C10 alkane (red trace in Fig. 4A) showing their relative positions at selected simulation time points. Native C18 FA after relaxation of the crystal structure (PDB entry: 6ZH7; resolution: 2.0 Å) is shown for comparison (light red).

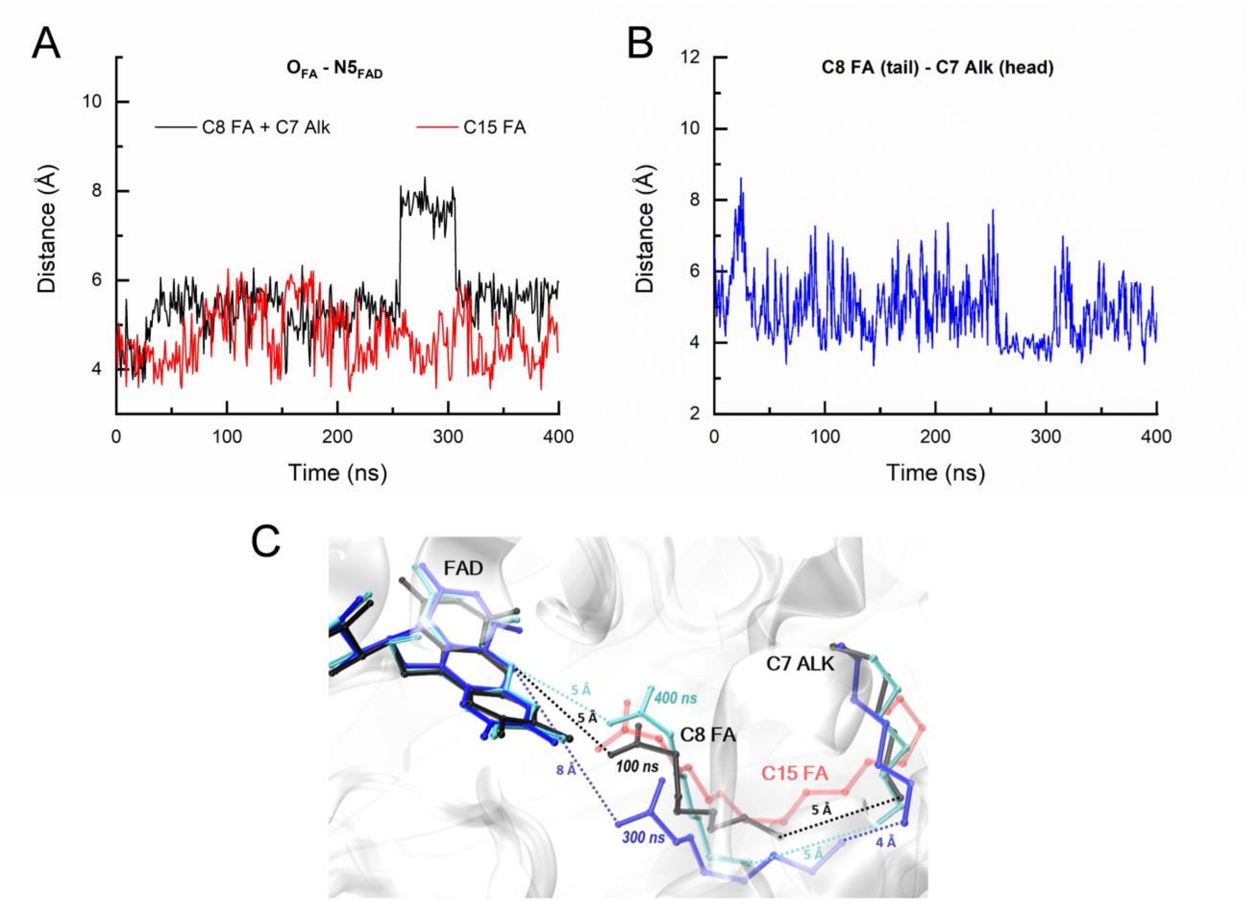

Fig. S4. MD Simulations of *Cv*FAP containing C8 FA + C7 alkane *vs.* C15 FA. Dynamics of the distances between: (**A**) the carboxyl O atoms of C8 FA and/or C15 FA and the N5 atom of the FAD isoalloxazine ring, and (**B**) the tail of C8 FA and the head of C7 alkane (carbon atom closest to C8 FA) in the course of the corresponding MD simulations. (**C**) Snapshots from the simulation of C8 FA + C7 alkane. The corresponding C15 after relaxation is shown for comparison (light red).

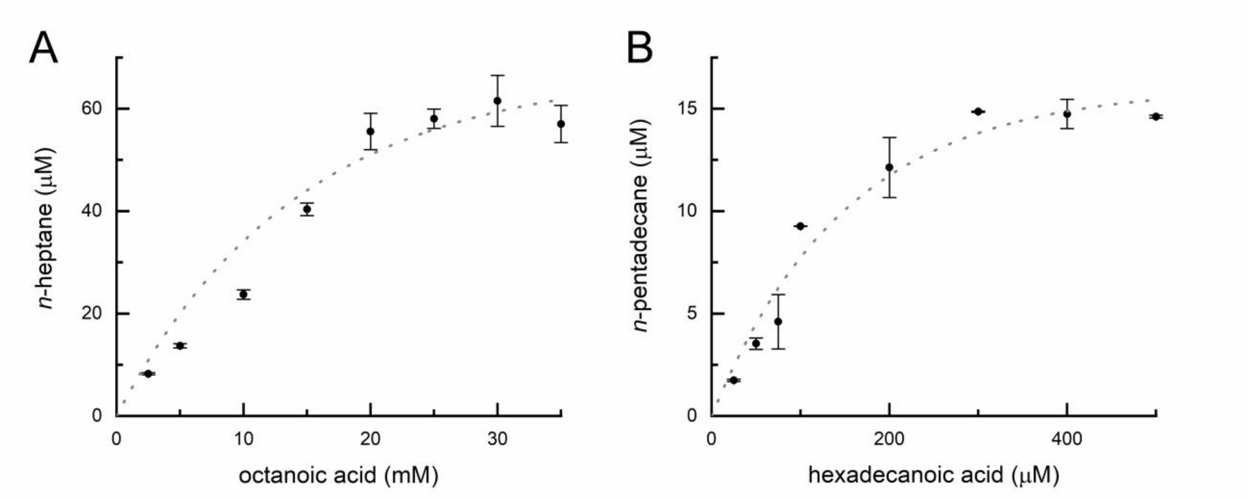

Fig. S5. Search for the *in-vitro* concentration optima/saturation levels for C8 and C16 FAs. (**A**) Yield of *n*-heptane as a function of added C8 substrate in the *in-vitro* activity test at pH 6 (optimum pH for C8 FA). (**B**) Yield of *n*-hexadecane as a function of added C16 substrate in the *in-vitro* activity test at pH 8.5 (optimum pH for C16 FA). *Cv*FAP concentration in these tests was ~70 nM. The samples were exposed to continuous light from a blue LED (emission maximum centered at 450 nm) of the intensity of 360 µmoles photons/m^2/s. Near-saturation concentrations of the individual substrates were used for the final comparison experiment shown in Fig. 5A.The error bars show the standard deviation obtained performing the experiments using three independent samples.
